# Complete Genomic Sequence of Bacillus coagulans Strain JBI-YZ6.3: a Natural Spore-Forming Isolate from Food-Grade Tapioca Starch

**DOI:** 10.1128/mra.01003-22

**Published:** 2022-12-06

**Authors:** Yongmei Zhang, Neil N. Gandhi

**Affiliations:** a Jeneil Biotech, Inc., Saukville, Wisconsin, USA; DOE Joint Genome Institute

## Abstract

Bacillus coagulans strain JBI-YZ6.3 is a safe probiotic bacterium isolated from food-grade tapioca starch. The complete genome of *B. coagulans* JBI-YZ6.3 comprises one circular chromosome of 3.5 Mb and contains no toxigenic and antibiotic resistance genes, providing molecular information to support the strain’s safety and usage as a probiotic.

## ANNOUNCEMENT

Bacillus coagulans is a spore-forming, lactic-acid-producing bacterium recognized widely for its probiotic activities (1). *B. coagulans* is ubiquitous in soil, and natural isolates have been found in various food products, including canned tomatoes, fermented soybeans, and dry milk powders (2, 3). *B. coagulans* is a safe probiotic bacterium that has been designated generally recognized as safe (GRAS) for human consumption (4). The ability of *B. coagulans* to sporulate provides inherent phenotypic benefits for surviving industrial processing conditions (thermal processing and low moisture) and the gastrointestinal tract (low pH and bile salts) (4, 5). Here, we report the closed genome of *B. coagulans* strain JBI-YZ6.3 to further elucidate the genetic basis for the strain’s beneficial phenotypic characteristics.

*B. coagulans* strain JBI-YZ6.3 was isolated from food-grade tapioca starch (manufactured in Thailand) in August 2020. A starch sample (1 g) was suspended in 0.3% peptone solution (10 mL) and heat treated at 85°C for 25 min. The heat-treated suspension (1 mL) was mixed with 4 mL of 1% agar (50°C), poured over glucose yeast extract (GYE) medium agar, and incubated at 45°C for 1 day. A single colony was selected and identified by 16S rRNA sequencing as *B. coagulans*, also known as Weizmannia coagulans (6). *B. coagulans* strain JBI-YZ6.3 has been deposited in ATCC (PTA-127366).

High-molecular-weight genomic DNA of strain JBI-YZ6.3 was purified using the Qiagen genomic tip kit. Library preparation, whole-genome sequencing, and *de novo* genome assembly were conducted at Azenta/Genewiz (South Plainfield, NJ). The DNA sample was quantified using a Qubit 2.0 fluorometer (Life Technologies, Carlsbad, CA) and checked for purity and integrity using Nanodrop and Agilent TapeStation instruments, respectively. The PacBio SMRTbell library (~20 kb) was constructed using the SMRTbell express template prep kit 2.0 (PacBio, Menlo Park, CA), bound to polymerase using the Sequel Binding kit 3.0 (PacBio), and loaded onto a PacBio Sequel instrument using Sequel sequencing kit 3.0. Sequencing on a PacBio Sequel SMRT cell produced a total of 8.33 Gb of data in 979,653 subreads (mean length, 9,210 bp). Read lengths displayed a typical distribution pattern of PacBio sequencing, with an average subread length of 8.5 kb and max subread length of >117 kb.

The quality of the demultiplexed subreads was checked; coverages were estimated. The subreads were assembled *de novo* using Canu v1.7 with default settings except for coverage cutoff set at 40-fold. The draft assembly was reviewed and further refined by merging contigs and dropping contigs that were potential artifacts from the assembly process using Canu v1.7 with default parameters. The merged contigs were checked, circularized, and reordered, starting with the *dnaA* gene using Circulator v2.3.2 with default settings. The assembled, circularized contig was polished with raw subreads using Arrow v2.3.2 with default parameters, yielding a single chromosome containing 3,509,359 bp with a G+C content of 46.34%. The average genome coverage was 2,019-fold.

The fully assembled genome was annotated using the PATRIC RASTtk-enabled Genome Annotation Service (7). A total of 3,942 protein-coding sequences (2,514 have functional assignments), 84 tRNAs, 27 rRNAs, and 2 CRISPR arrays were predicted. Over 80 genes were annotated with functions in sporulation. Over 100 genes were predicted in the B-group vitamin biosynthesis. No toxigenic genes were found in JBI-YZ6.3 genome. The Similar Genome Finder (PATRIC) identified *B. coagulans* strain DSM 2314 as the most similar genome, with a k-mer count at 617/1,000 (distance, 0.0129), demonstrating that JBI-YZ6.3 is a unique strain. Genome-wide comparative studies of JBI-YZ6.3 with other *B. coagulans* strains will provide new insights on the molecular basis of its beneficial probiotic activities (8–10).

### Data availability.

This genome project is indexed at GenBank (BioProject PRJNA879771). The complete genome sequence of *B. coagulans* strain JBI-YZ6.3 is deposited in GenBank (CP104390). The Sequence Read Archive (SRA) accession number is SRR21643318.
